# Experimental Study on the Flow Regimes and Pressure Gradients of Air-Oil-Water Three-Phase Flow in Horizontal Pipes

**DOI:** 10.1155/2014/810527

**Published:** 2014-01-12

**Authors:** Luai M. Al-Hadhrami, S. M. Shaahid, Lukman O. Tunde, A. Al-Sarkhi

**Affiliations:** ^1^CER/Research Institute, King Fahd University of Petroleum and Minerals, Dhahran 31261, Saudi Arabia; ^2^Department of Mechanical Engineering, King Fahd University of Petroleum and Minerals, Dhahran 31261, Saudi Arabia

## Abstract

An experimental investigation has been carried out to study the flow regimes and pressure gradients of air-oil-water three-phase flows in 2.25 ID horizontal pipe at different flow conditions. The effects of water cuts, liquid and gas velocities on flow patterns and pressure gradients have been studied. The experiments have been conducted at 20°C using low viscosity Safrasol D80 oil, tap water and air. Superficial water and oil velocities were varied from 0.3 m/s to 3 m/s and air velocity varied from 0.29 m/s to 52.5 m/s to cover wide range of flow patterns. The experiments were performed for 10% to 90% water cuts. The flow patterns were observed and recorded using high speed video camera while the pressure drops were measured using pressure transducers and U-tube manometers. The flow patterns show strong dependence on water fraction, gas velocities, and liquid velocities. The observed flow patterns are stratified (smooth and wavy), elongated bubble, slug, dispersed bubble, and annular flow patterns. The pressure gradients have been found to increase with the increase in gas flow rates. Also, for a given superficial gas velocity, the pressure gradients increased with the increase in the superficial liquid velocity. The pressure gradient first increases and then decreases with increasing water cut. In general, phase inversion was observed with increase in the water cut. The experimental results have been compared with the existing unified Model and a good agreement has been noticed.

## 1. Introduction

Multiphase flow occurs in oil/gas, chemical, civil, and nuclear industries. The dominant occurrence of gas-oil-water three-phase flow in the petroleum industry requires sound knowledge of the behavior of multiphase flow. The most important characteristic of multiphase flow is its flow pattern (physical distribution of the phases within the enclosure they flow through) and the pressure gradient along the horizontal pipeline. In this regard, it is imperative to fully understand and study the flow rates, flow regimes/patterns, liquid-hold-up/water cut (WC), pressure gradients, and volume fractions of gas, oil, and water going into the pipelines during transportation of petroleum products. The water cut (WC) is the water quantity at the pipe inlet as volume percentage of the total inlet volumetric flow rate. The water cut is always the basis for pipelines and equipment design. During the transportation of the multiphase flow, water in the system starts separation and thereby accumulates at the pipe bottom and that amount of water is being referred to as local water contents, local water, or water hold-up. Also, it is important to better understand/predict/investigate the flow characteristics during petroleum production at different flow conditions such as the geometrical configuration of the pipeline, the physical properties of the fluids, and flow rates. There is a need to accurately investigate and predict the flow configurations and the pressure drop [1, 2].

The presence of water, salts, and carbon dioxide gas in petroleum products is the main cause of carbon steel pipelines corrosion during oil transportation and storage. At low water cut, the corrosive water does not create problems when water is fully dispersed in oil. Most oil wells operate at different water cuts, as high as 90%, which lead to different flow regimes. As water cut increases, water droplets start to coalesce and phase separation of oil and water occurs. In horizontal or near horizontal pipes, the three-phase flow along the pipe with air flows at top of the pipe, oil flows at the middle and water flows at the bottom of the pipe due to difference in densities. Each phase wets parts of the pipe. The possibility of corrosion is high when water phase is in contact with the pipe wall. It is therefore important to understand the three-phase air-oil-water behavior in production pipelines and also predict the flow patterns, pressure gradient, and consequently controlling the pipe corrosion. Several studies have been carried out on characteristics of oil-water-gas [3–24].

Sobocinski [3] performed experimental research on the three-phase water-air and diesel oil and air in a 7.62 cm internal diameter transparent horizontal plastic pipe. He carried out 114 tests to observe flow pattern and measure pressure drop and hold-up of the three-phase air-oil-water. This is one of the earliest research works on multiphase flow. Malinowsky [4] carried out experimental study on three-phase air-oil-water flow in a horizontal pipe. A total of 34 tests were conducted in a 1.5-inch inner diameter transparent acrylic pipe to measure the pressure gradients. He compared his experimental results with that of Beggs and Brill [5] and that of Duckler et al. [6].

Laflin and Oglesby [7] conducted 79 experiments on air-oil-water three-phase flow. Flow rates and pressure gradients were recorded while the flow patterns were plotted on those of Beggs and Brill [5] and Mandhane et al. [8]. Their data was in the flow regime of intermittent flow and they also investigated flow rates near the inversion point. Stapelberg [9] carried out experimental study on three-phase gas, water, and mineral oil experiments in 23.8 mm and 59 mm internal diameter (ID) horizontal pipes. The viscosity of the oil was 31 centipoise (cp) and the flow regimes of stratified and slug flow were studied while also measuring the pressure gradients, slug lengths, slug frequency, and other slug characteristics. New data were provided and inadequacy of methods used for calculating pressure gradient especially in stratified three-phase flows was also demonstrated.

Açikgöz et al. [10] performed experiments on the three-phase air-water and mineral oil (with 864 kg/m^3^ density and viscosity of 0.1164 Pa.s.) in a horizontal pipeline by observing the flow regimes and also constructed flow regime maps. The flow regime map was constructed by keeping the oil superficial velocity constant, increasing the water superficial velocity slowly and also keeping the air superficial velocity constant so as to determine the transition point from oil to water based flow. The same technique was used to acquire data for the flow regime transition points. The three-phase flow regime was classified into ten groups.

Hall [11] carried out experimental study on gas-oil-water three-phase flow in horizontal pipes. He modeled the three-phase stratified flow by using the obtained hold-up to calculate the transition from stratified flow to slug flow. The model was compared with experimental data which showed that the transition occurred at higher gas velocities than those predicted by the model. The oil layer was believed to be the reason, because it travels at a higher mean velocity since its lower interface was in contact with a moving water layer and not a fixed wall.

Lahey et al. [12] performed experiments in a 19 mm inner diameter pipe using three-phase fluids of air, water, and mineral oil with viscosity of 116 cp. Flow patterns were observed while oil hold-up and water hold-up were measured. It was observed that the region of the stratified flow for the small diameter was very restricted.

Donnelly et al. [13] performed two and three-phase air/water and air/oil/water experiments, respectively, in a 25.9 mm inner diameter pipe. Several flow patterns were observed while pressure drop and hold-up were also measured for each system. Flow regime map was formulated and modifications to the momentum balance for the prediction of three-phase pressure gradient and phase slippage were also suggested.

Ajay [14] conducted two and three-phase flows in a water-oil-gas horizontal flow system. The experiments were performed in a 10.16 cm ID, 10 m long plexi-glass pipeline with a 2 m long plexi-glass test section. Flow patterns were observed, pressure gradients were measured and compared with results from previous work, and good agreement was reached. It was observed for the stratified oil-water-gas three-phase flows that the total liquid film height increases with increasing total liquid velocity but decreases with increasing gas velocity.

Hold-ups of stratified three-phase flow pattern of gas-oil-water was calculated by Taitel et al. [15]. Three steady state solutions for the upward inclined case were obtained. The only stable configuration was the one with the thinnest liquid layer. The essential step for the calculation of the hold-up, pressure drop, and transition criteria of the flow pattern was found to be the information regarding the liquid and oil levels in the pipe.

Chen and Guo [16] investigated flow patterns and pressure drop of air-oil-water in two different helically coiled tubes with ID of 39 mm and coil diameters of 265 mm and 522.5 mm, respectively. Flow patterns were observed for both two-phase oil-water and three-phase air-oil-water. The flow patterns were classified into four different regimes in each case. Flow pattern transition criteria equations were deduced from the experimental data and the equations showed good agreement when compared with the experimental data. A modified Chisolm correlation was presented in order to predict the pressure drop of gas-oil-water three-phase flow in horizontal coiled tubes.

Badie et al. [17] carried out experiments in an axial viewing system of a 37 m long, 78 mm ID test section using oil, water, and air. The effects of the entrained liquid flows on high gas velocities were studied. It was observed that the entrained liquid phase in the gas core was mainly due to intermittent bursting of waves at the bottom of the pipe. Oddie et al. [18] conducted two and three-phase flow experiments in a transparent 11 m long, 15 cm inner diameter pipe using kerosene, tap water, and nitrogen. 444 tests were conducted for observing different flow patterns and measuring hold-up. The flow pattern and hold-up were compared with the prediction of a mechanistic model of Petalas and Aziz [19] and the results gave good agreement.

Spedding et al. [20] carried out experiments on two different horizontal three-phase oil-water-air experimental setups. The ID of the two set up was 25.9 mm and 50.1 mm in which measurements and observations were taken in a 2 m length set between the 1.7 m outlet section and 4 m inlet section for the first facility. For the second facility with the 50.1 mm internal diameter has a 4.52 m test section set between 2 m outlet and 6 m inlet. 22 flow regimes that is, broadly classified into oil dominated and water dominated, were described in the work. A new type of flow regime mapping scheme was also presented to successfully predict two and three-phase systems.

Zhang and Sarica [21] developed a model called unified model to predict the flow pattern and pressure gradient of three-phase gas-oil-water which was an improvement on the earlier unified model of Zhang et al. [22]. The model was compared with experimental measurements of three-phase gas/oil/water pipe flows. The three-phase unified model gave better predictions than the unified model of gas/liquid two-phase pipe flow when compared with the experimental measurements of Khorr [22] for stratified gas/oil/water flow in horizontal and 1.5° downward pipes. Similar performance was seen when the two models were also compared with the experimental measurements of Hall [11] on pressure gradients for three-phase slug flow in a horizontal pipe.

Adrian Wegmann et al. [23] carried out three-phase oil-water-air experiment using paraffin oil, deionized water, and air for 5.6 mm and 7 mm ID pipes. Six flow patterns were observed and flow pattern maps were built for both pipes. The flow pattern maps were built with a constant air superficial velocity (VSA) by varying both paraffin and water superficial velocity for each map. Different cases of flow pattern maps were built for the VSA range from 0.2 m/s to 6.77 m/s. There was no agreement when the experimental data were compared with existing three-phase flow maps which might be due to the geometrical configuration of the set-up and physical properties of the fluids being used, but there was good match when compared with the theoretical transition boundary of Taitel et al. [15] in which the low viscosity ratio may be the reason.

Wang et al. [24] performed experiments on high viscosity oil/water/gas three-phase flows in a 2.067-inch ID pipe. The test fluids used were oil within 150 cp and 570 cp viscosity, filtered tap water, and natural gas. The flow patterns and slug characteristics were observed and pressure gradients and liquid hold-up were measured. The experimental results were compared with the unified model predictions of Zhang and Sarica [21] and the differences were noted.

The above work indicates that no study has been conducted to examine the cocurrent flow characteristics of air-water-Safrasol D80 oil which has a viscosity close to that of water as compared to the available in literature in horizontal acrylic pipe (with 2.25 cm inner diameter). The objective of the present investigation includes experimental study focusing on the flow regimes and pressure gradients of air-oil-water three-phase flows in horizontal acrylic pipe (with 2.25 cm ID) at different flow conditions. The experiments have been conducted at room temperature of 20°C using safrasol D80 oil density 800 kg/m^3^ and dynamic viscosity of 1.77centi-poise, tap water with dynamic viscosity of 1 centi-poise and 1000 kg/m^3^ density and air with dynamic viscosity of 0.000018 Pa s and 1.3 kg/m^3^ density. Superficial water and oil velocities varied from 0.3 m/s to 3 m/s and gas/air velocity varied from 0.29 m/s to 52.5 m/s in order to cover wide range of flow patterns. The experiments were performed for 10% to 90% water cut (WC) in steps of 10%. The flow patterns were observed and recorded using high speed video camera (hp CW450t) while the pressure drop was measured using pressure transducers and U-tube manometers. The effects of water cut, liquid velocity, and gas velocity on pressure drop and flow patterns have been studied systematically. Knowledge of the above parameters is essential because most oil wells operate at different water cuts, as high as 90%, which leads to different flow regimes.

## 2. Description of the Experiment

**Figure 1:**
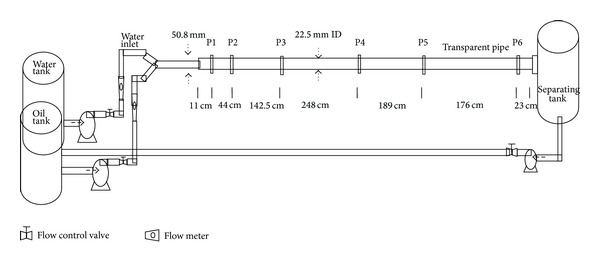
Schematic layout of the air-oil-water three-phase flow loop.

The schematic of the gas-oil-water horizontal three-phase flow loop is depicted in Figure 1. The experiments were conducted under controlled room temperature of average of 20°C. The single phase water was pumped first using a rotameter via a 2.2 KW, 3 hp centrifugal pump to the horizontal pipeline. Then, the oil was also pumped into the pipeline and they both combined at the Y-section of the PVC pipe. The air was then mixed with the combined oil-water through a hose connected to the pipeline. The three-phase fluids (air-oil-water) then flow simultaneously to the acrylic pipe along the test section. The manometer was connected to the pressure taps along the test section to measure the pressure drop and also the flow patterns were observed. The three-phase fluids were then discharged into the slug catcher tank from the test section after which they were dumped into the separating tank. The separating tank and slug catcher have openings to allow the gas to escape to the atmosphere while the oil and water separate under gravity due to density differences in the separating tank. The oil and water were then returned to their original tanks through another pump connected to the separating tank. The loop process was repeated again till all the experiments were conducted.

The oil and water were stored inside separate tanks. Four tanks were used for the experiments. One was used for oil and another one was used for water while the remaining two were used as slug catcher tank and separating tank, respectively. The tanks are made of fiber-glass with volume capacity of 1200 liters each. The air compressor is the Kaeser compressor air center SM-12 manufactured by Kaeser Compressor Inc. It has an integrated refrigerated air dryer to avoid moist air inside the system and it also has variable speed drive to regulate the air flow rate inside the pipeline. The controlled pressure capacity of the air storage tank is 7 bar. The loop system has two alternative rotameters (for oil and water) each made from King Instrument Company. The first rotameter covers lower volumetric flow rate range 1–10 gpm with an error of ±3% while the second rotameter covers higher volumetric flow rate range 4–40 gpm with an error of ±6%. The maximum flow rate obtained for water was 23 gpm while that of oil was 21 gpm. The air flow meter is manufactured by Omega to measure the air flow rate that goes into the pipeline with capacity range from 0 to 338 gpm. The multiphase flow loop has three 2.2 KW and 3 horse-power centrifugal pumps manufactured by Crompton Greaves Ltd. Two of the centrifugal pumps were used to pump the oil and water each from their respective tank while the third pump was used to pump the oil and water from the separating tank back to their original tanks controlled through a control panel. The flow loop has a mercury U-tube manometer and a pressure transducer made from Rosemount Company to measure the pressure drop along the pipeline.

**Figure 2:**
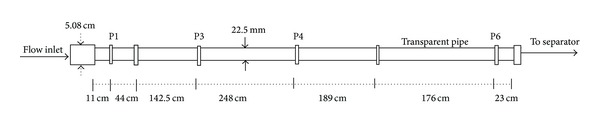
Schematic layout of the air-oil-water test section.

The test section is 8.33 m long with internal diameter (ID) of 2.25 cm (with L/D = 370) with an entrance diameter of 5.08 cm as shown in Figure 2. The three-phase air-oil-water enters the test section via the 5.08 cm entrance diameter which is then reduced to the 2.25 cm ID in which the three-phase fluids flow till they discharge to the slug catcher tank. The test section has six pressure taps from P1 to P6 where the manometer and pressure transducer were connected. The test section also consists of a 2.75 cm ID and 136 cm long transparent pipe. The transparent pipe was used to visualize the flow pattern while the U-tube mercury manometer was connected at pressure taps P3 and P6 to measure the pressure drop while the pressure drop was also measured from the differential pressure transducer. The distances between the pressure taps are shown in Figure 2. A high speed hp CW450t digital camera was also placed at 0.5 m perpendicular to the pipeline to record the flow patterns with shutter speed of 1/250.

## 3. Experimental Procedure

The oil tank was filled with Safrasol D80 and the water tank was filled directly from the main supply of tap water through a rubber hose while the air compressor was switched on in order to fill it with air with pressure rating of 7 bar.

A fully developed flow was achieved before all the experimental was recorded. Constant-Machado et al. [25] reported that a single phase fully developed flow can be reached at the distance of 50–100 pipe diameters at the low Reynold's number (Re) of 2500. For the multiphase flow, Jepson [26] proved that a fully developed flow could be achieved at a pipe length less than 50 pipe diameters at a relatively high Re due to the interaction of the different phases. In this context, for the present test section with 0.0225 m ID, L/D of 370, and for the velocity range of 0.2–3 m/s, a fully developed single phase water flow with Re between 11,777 and 100,000 with oil single phase and air single phase could be achieved at less than 1.8 m from the inlet.

Additionaly, for air-oil-water flow, a fully developed turbulent flow can be achieved at less than 1.2 m from the y mixing section. Since the distance between this y-section and the first pressure tap is around 1.87 m, then a fully developed flow can be achieved easily before taking measurements.

The pressure drop displayed in inches of water in the transducer was recorded while the difference in height of mercury in the U-tube manometer was also recorded. Once stabilized, pressure readings and flow are achieved in the manometers and transducers and pressure drop and flow patterns were then recorded for all experiments. The error in the manometer is 0.05 inch Hg. The experiments were performed under full pipe flow conditions. Videos of the flow regime were taken and the air-oil-water flow rates were varied for all the experiments. As soon as the oil or water in the initial tanks was exhausted, all pumps were switched off and oil and water were left in the settling tank while the air escapes from the top openings of the tanks in order to allow enough time for the oil and water to separate. Finally, the separating tank valves were opened in order to allow the water to be recycled first to its tank since it will be at the bottom due to its higher density, followed by the oil. The mixed oil and water were dumped in the drain. The process was repeated all over again till all the experiments were completed. Several experiments were performed in order to observe/cover all flow patterns. The matrix range for three-phase flow of air-oil-water experiments is shown in Table 1. The effects of water cut, liquid velocity, gas velocity, pressure drop, and flow patterns were studied systematically.

## 4. Results and Discussion

The experiments were carried out in an acrylic pipe to visualize the flow patterns. The test fluids used were Safrasol D80 oil, tap water and air (properties of these fluids are mentioned earlier in Introduction). The three different fluids were passed into the horizontal pipeline and the flow patterns were observed while the pressure gradients were measured/recorded (using pressure transducers and U-tube manometers). A total of 377 data points were acquired and studied. The matrix range for three-phase flow of air-oil-water experiments is shown in Table 1. The effects of water cut, liquid velocity, gas velocity on flow patterns, and pressure drop have been studied.

### 4.1. Effect of Water Cuts, Liquid, and Gas Velocities on Flow Patterns

This is the geometric configuration of the gas and liquid phases in the pipe. The flow configurations differ from each other in the spatial distribution of the interface. In order to achieve more accurate modeling of the flow and also to have a better understanding of the phenomena occurring during the gas-liquid phase flow, it is important to recognize the boundaries between flow patterns. Collier and Thome have discussed various types of flow patterns of multiphase flows [27]. Although, lot of research studies are presented (in literature) on flow pattern maps of air-oil-water, but no work has been reported on the flow pattern map of air-oil-water in a horizontal acrylic pipe with 0.0275 m ID. It is necessary to identify the different flow patterns of the horizontal cocurrent flow of air-oil-water to examine the effect of water cuts on flow patterns.

**Figure 3:**
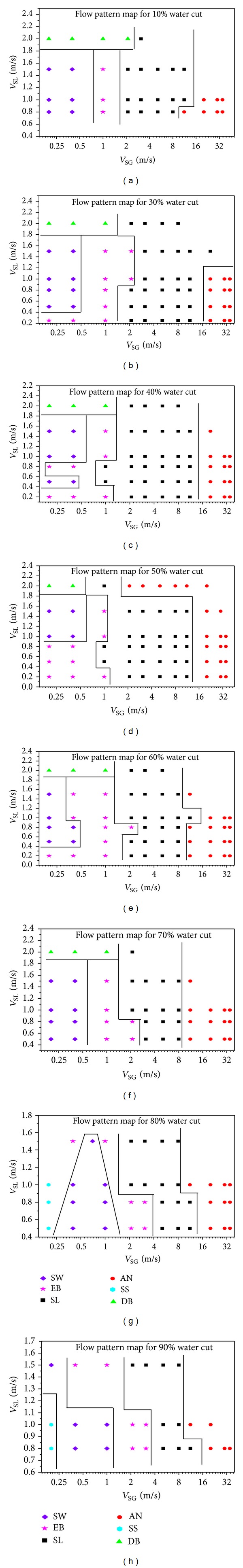
Flow pattern maps of Air-Oil-Water for different water cuts (10%–90%) and for different superficial liquid (*V*_SL_ ) and gas velocities (*V*_SG_).

The resultant flow pattern data for the air-oil-water flow are plotted in Figure 3 for 10% to 90% water cut. The superficial liquid velocity ranges from 0.2 m/s to 2 m/s while the superficial gas velocity ranges from 0.20 m/s to 35.14 m/s. As it can be seen from figures, in all the water cuts (0.1–0.9), six different flow patterns (with only five flow patterns present in each water cut) were observed for cocurrent air-oil-water flow in a horizontal acrylic of 0.0275 m ID pipe. These flow patterns are stratified (smooth and wavy), elongated bubble, slug, dispersed bubble, and annular flow patterns. The results show strong dependence of flow patterns on water fraction, gas velocities, and liquid velocities The superficial liquid velocity *V*_SL_ is the sum of the superficial oil velocity *V*_SO_ and superficial water velocity *V*_SW_ (i.e., *V*_SL_ = *V*_SO_ + *V*_SW_).

For the 10% water cut (for *V*_SL_ up to 1 m/s), it started with stratified wavy until the final transition to annular flow pattern. For *V*_SL_ = 1.5 m/s, the transition slug flow could not be seen, while for high *V*_SL_ of 2 m/s, dispersed bubble flow pattern appears and it transits to slug flow. This trend continues till 40% water cut. It was noticed that as the superficial liquid velocity increases, the flow pattern changes from elongated bubble to stratified wavy and finally to dispersed bubble for 50% water cut at lower superficial gas velocity, but for higher superficial velocity, it was noticed that as the *V*_SL_ increases, the flow pattern changes from slug flow to annular flow pattern. The 60% water cut is also similar to 50% water cut in flow pattern transition.

For 80% and 90% water cut, a stratified smooth flow pattern was observed, but dispersed bubble flow pattern was absent in both water cuts unlike in the previous water cuts where stratified smooth pattern was absent with the presence of dispersed bubble. Finally, at very high superficial gas and liquid velocity, the flow patterns were mostly annular flow pattern for all the water cuts.

There is no generalized flow pattern map for air-oil-water flow in pipelines since the flow pattern in the system depends on the physical properties of the fluids and the wetting properties of the wall surface. High pressure gradient has been observed at annular flow pattern than in any other flow patterns. The flow pattern was compared with unified model [22] and it gave good results.

### 4.2. Effect of Water Cuts, Liquid, and Gas Velocities on Pressure Gradients

**Figure 4:**
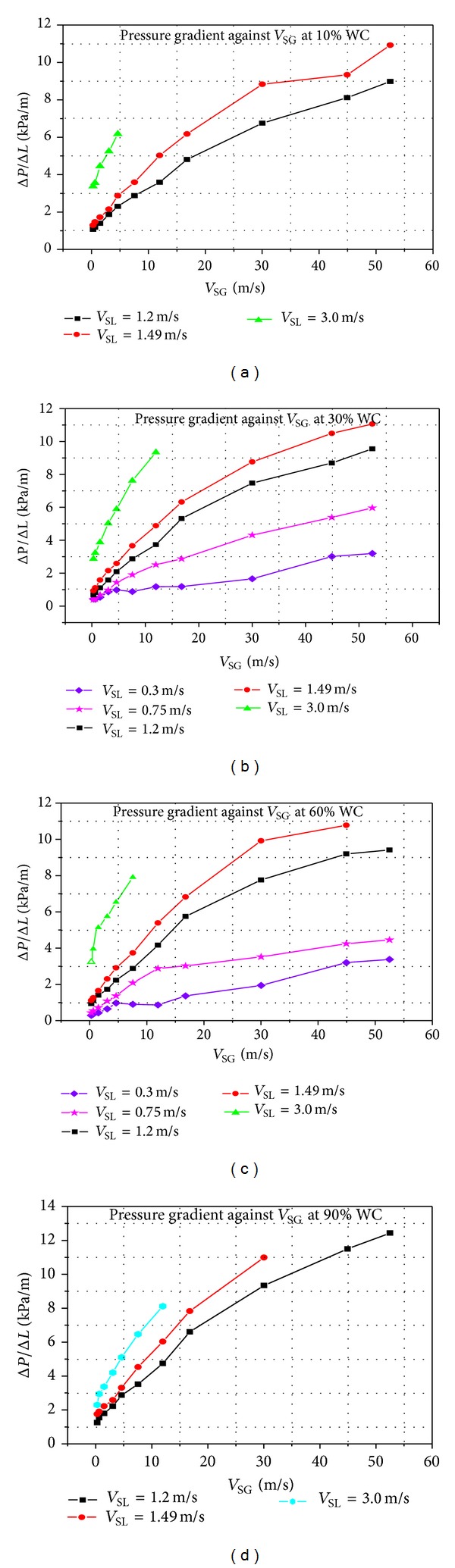
Effect of superficial gas velocities on pressure gradients for different water cuts and superficial liquid velocities.

**Figure 5:**
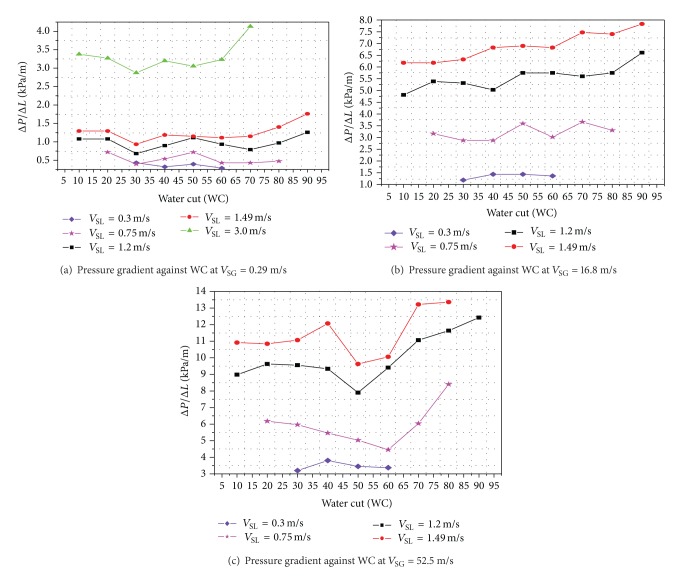
Effect of water cuts on pressure gradients for different superficial gas velocities and superficial liquid velocities.

The pressure gradients of cocurrent air-oil-water flow in a horizontal acrylic pipe for superficial liquid velocities (*V*_SL_) between 0.3 m/s and 3 m/s and superficial gas velocities (*V*_SG_) between 0.29 m/s and 52.5 m/s and water cuts from 0.1 to 0.9 are presented in Figures 4 and 5. The first pressure tap was fixed at 1.87 m (to ensure that the flow is fully developed) from the pipe inlet in order to have accurate pressure measurement.

The pressure gradients increase with increase in *V*_SG_ and *V*_SL_. The effects of different factors on the pressure gradients include the effect of *V*_SG_ at different water cuts for varying *V*_SL_, the effect of water cuts at different *V*_SG_ for varying *V*_SL_, the effect of liquid mixture Reynold's number and the effect of *V*_SL_ at different water cuts, and so forth that will be explained in the preceding sections.

#### 4.2.1. Effect of Superficial Gas Velocities, *V*_SG_, on Pressure Gradients

The pressure gradient increases with increasing gas flow rates. The increase in *V*_SG_ led to transition of flow pattern in which the pressure gradient is the highest for annular flow and the lowest for stratified and dispersed bubble flow pattern. For a particular *V*_SG_, as the superficial liquid velocity increases, the pressure gradient also increases.

The effect of superficial gas velocities (0.29 m/s to 52.5 m/s) on pressure gradients for different water cuts (10%, 30%, 60% 90%) and different superficial liquid velocities (0.3 m/s, 0.75 m/s, 1.2 m/s, 1.49 m/s, 2.24 m/s and 3.0 m/s) is shown in Figure 4.

It can be seen in Figure 4 that the pressure gradients increase with increasing gas and liquid flow rates. For 10% water cut, there were no large changes in pressure gradient for *V*_SG_ between 0.29 m/s and 0.63 m/s, and the maximum difference between *V*_SL_ of 1.2 m/s and 1.49 m/s was 107.77 Pa/m and 179.61 Pa/m, respectively. This is obvious since the flow patterns at these *V*_SL_ were stratified.

At higher *V*_SG_, the situations were different and the pressure gradients were affected clearly by increasing *V*_SG_ and the effect became pronounced by increasing *V*_SG_ and *V*_SL_. For *V*_SG_ more than 16 m/s, the pressure gradients were higher. For *V*_SL_ of 1.2 m/s and 1.49 m/s, the pressure gradients were 8.98 kPa/m and 10.92 kPa/m, respectively, at 52.5 m/s *V*_SG_. This is due to the fact that the flow patterns were mainly annular flow pattern. The 90% water cut was a little bit similar to that of 10% water cut with the exception of a maximum attained new *V*_SL_ of 2.24 m/s in 90% water cut. Similar trends were observed for different water cuts and *V*_SL_.

*V*_SL_ of 0.3 m/s, 0.75 m/s, 1.2 m/s, 1.49 m/s, 2.24 m/s, and 3.0 m/s.

#### 4.2.2. Effect of Water Cut and *V*_SL_ on Pressure Gradients

The pressure gradient first increases and then decreases with increasing water cut. The effect of increasing water cut usually leads to phase inversion. For a particular water cut, as the superficial liquid velocity increases, the pressure gradient also increases.

The effect of water cuts (10 to 90%, in steps of 10%) on pressure gradients for different superficial gas velocities (0.29 m/s, 16.8 m/s and 52.5 m/s) and different superficial liquid velocities (of 0.3 m/s, 0.75 m/s, 1.2 m/s, 1.49 m/s, and 3.0 m/s) is shown in Figure 5.

The pressure gradient for *V*_SG_ of 0.29 m/s at *V*_SL_ of 0.3 m/s, the maximum peak of pressure gradient 432 Pa/m, was at 0.3 water fraction while the minimum pressure of 287.4 Pa/m, was at 0.6 water fraction. For 0.75 m/s *V*_SL_, the pressure gradients started at 0.2 water fraction with its maximum peak of 718.4 Pa/m while the minimum pressure of 395.1 Pa/m was at 0.3 water fraction. The *V*_SL_ was increased to 1.2 m/s, maximum peak of 1.26 kPa/m at 0.9 water fraction the maximum peak of pressure gradient 1.1 kPa/m at 0.5 water fraction, while the minimum pressure of 682.5 Pa/m was at 0.3 water fraction. For *V*_SL_ of 1.49 m/s, the maximum peak of pressure gradient 1.76 kPa/m was at 0.9 water fraction while the minimum pressure of 934 Pa/m was at 0.3 water fraction. The *V*_SL_ was increased to 3 m/s and the maximum peak of pressure gradient was found to be 4.13 kPa/m at 0.7 water fraction while the minimum pressure gradient of 2.87 kPa/m was at 0.3 water fraction. The above procedure was repeated for different *V*_SG_ (0.63, 1.51, 3.07, 4.62, 7.56, 12.0, 16.8, 30.0, 44.9, and 52.5 m/s).

**Figure 6:**
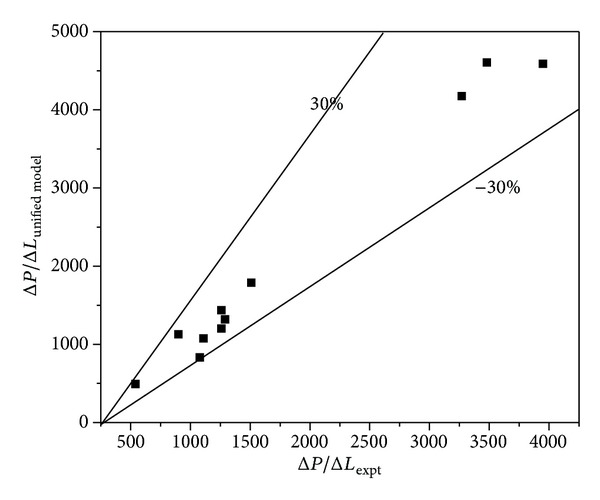
Comparison of the experimental results with the Unified Model of Zhang and Sarica [21].

Similar type of behavior (as explained above) has been observed for different *V*_SG_. In general, it has been noticed that for a particular *V*_SG_, as the *V*_SL_ increases, the maximum pressure gradient also increases.  Figure 6 also shows the effect of water cuts on pressure gradients for different *V*_SL_ and for *V*_SG_ = 16.8 m/s and 52.5 m/s. For *V*_SG_ =16.8 m/s, the pressure gradients at higher values of *V*_SL_ (i.e., at 3.0 m/s) could not be observed due to high *V*_SG_.

The pressure gradient from the experimental data was compared with the unified model [22]. It was discovered that, at low *V*_SG_ of 0.29 m/s and 0.63 m/s, the results were in good agreement with maximum error of 30% for all *V*_SL_. However, experimentally measured pressure gradients did not exhibit good comparison at high *V*_SG_ for all levels of *V*_SL_. The comparison is shown in Figure 6 and Table 2. As it can be seen, the flow patterns were compared with unified model and they gave good results.

## 5. Conclusions

The present experimental investigation has discussed in appreciable depth the flow regimes and pressure gradients of air-oil-water three-phase flows in a 2.25 ID horizontal pipe at different flow conditions. The effect of water cuts, liquid, and gas velocities on flow patterns and pressure gradients have been studied. Superficial water and oil velocities varied from 0.3 m/s to 3 m/s and gas/air velocity varied from 0.29 m/s to 52.5 m/s to cover wide range of flow patterns. The study was performed for 10% to 90% water cut. The experiments have been conducted using low viscosity oil Safrasol D80 oil, tap water, and air. The observed flow patterns show strong dependence on water fraction, gas velocities, and liquid velocities. The observed flow patterns are stratified (smooth and wavy), elongated bubble, slug, dispersed bubble, and annular flow patterns. The pressure gradients have been found to increase with increase in gas flow rates. Also, for a given superficial gas velocity, the pressure gradients increased with increase in the superficial liquid velocity. The pressure gradient first increases and then decreases with increasing water cut. In general, phase inversion was observed with increase in water cut. The experimental results have been compared with the existing unified model and a good agreement has been noticed.

## Figures and Tables

**Table 1:** The matrix range for three-phase flow of air-oil-water experiments.

*V*_SG_ (m/s)	*V*_SL_ = *V*_SO_ + *V*_SW_ (m/s)	Water cut (WC)
0.20–52.5	0.2–3.0	0.1–0.9 (in steps of 0.1)

**Table 2:** Comparison of the experimental results with the unified model.

*V*_SO_ (m/s)	*V*_SW_ (m/s)	*V*_SG_ (m/s)	FP_Unif Mod_	FP_Expt_	Experimental pressure gradient	Unified Model pressure gradient	% Absolute error
0.96	0.24	0.29	INT	ST	1080	833	22.84
1.192	0.289	0.29	D-B	D-B	1290	1319	2.22
2.4	0.6	0.29	D-B	D-B	3270	4174	27.63
0.3	0.45	0.63	INT	INT (SL)	540	493	8.62
0.48	0.72	0.63	INT	INT (EB)	1110	1075	3.20
0.598	0.892	0.63	INT	INT (EB)	1260	1436	14.00
1.2	1.8	0.63	D-B	D-B	3950	4588	16.14
0.96	0.24	0.63	INT	INT (EB)	1260	1201	4.76
1.192	0.289	0.63	INT	INT (EB)	1510	1789	18.48
2.4	0.6	0.63	D-B	D-B	3480	4606	32.35

Note: FP_Unif Mod_ refers to flow patterns of unified model; FP_Expt_ refers to flow patterns of present experimental work.

## Nomenclature

*A*: Cross-sectional area of the pipe
*A*_*a*_: Cross-sectional area of the pipe occupied by air
*A*_*o*_: Cross-sectional area of the pipe occupied by oil
*A*_*w*_: Cross-sectional area of the pipe occupied by water
AN: Annular flow pattern
cp: Centipoise
*D*: Diameter of the pipe
DB: Dispersed bubble flow pattern
EB: Elongated bubble flow pattern
*f*: Friction factor
FP_Expt_: Experimental flow pattern
FP_Unif Mod_: Unified model flow pattern
gpm: Gallon per minute
*H*_*L*_: Liquid hold-up
ID: Inner diameter
INT: Intermittent flow pattern
*L*: Length of the pipe
lpm: Liter per minute
*m*_*a*_: Mass flow rates of air
*m*_*o*_: Mass flow rates of oil
*m*_*w*_: Mass flow rates of water
*M*_total_: Mass flow rates of oil
*Q*_*a*_: Volumetric flow rates of air
*Q*_*o*_: Volumetric flow rates of oil
*Q*_*w*_: Volumetric flow rates of water
*Q*_total_: Total volumetric flow rates
*Re*: Reynold's number
*Re*_mixture_: Liquid mixture Reynold's number
SL: Slug flow pattern
SS: Stratified smooth flow pattern
ST: Stratified flow pattern
SW: Stratified wavy flow pattern
*V*_*a*_: Average in situ velocity of air
*V*_*o*_: Average in situ velocity of oil
*V*_*w*_: Average in situ velocity of water
*V*_Smix_: Superficial mixture velocity
*V*_SG_: Superficial velocity of gas
*V*_SO_: Superficial velocity of oil
*V*_Sw_: Superficial velocity of water
WC: Water cut.

### Greek Symbols

*ρ*_*a*_: Density of air
*ρ*_*o*_: Density of oil
*ρ*_*w*_: Density of water
Δ*P*: Pressure drop
Δ*P*/Δ*L*: Pressure gradient
(Δ*P*/Δ*L*)_*TP*_: Pressure gradient of three-phase air-oil-water
(Δ*P*/Δ*L*)_Water_: Pressure gradient of single-phase water
*ε*: Pipe roughness.

## References

[1] Brown KE (1977). *The Technology of Artificial Lift Methods*.

[2] Hashizume K, Ogawa N (1987). Flow pattern, void fraction and pressure drop of refrigerant two-phase flow in a horizontal pipe-III. Comparison of the analysis with existing pressure drop data on air/water and steam/water systems. *International Journal of Multiphase Flow*.

[3] Sobocinski DP (1955). *Horizontal Co-current flow of water, gas-oil and air [M.S. thesis]*.

[4] Malinowsky MS (1975). *An experimental study of oil-water and air-oil-water flowing mixtures in horizontal pipes [M.S. thesis]*.

[5] Beggs HD, Brill JR (1973). Study of two-phase flow in inclined pipes. *Journal of Petroleum Technology*.

[6] Duckler AE, Wicks M, Cleveland RG (1964). Frictional pressure drop in two-phase flow: a comparison of existing correlations for pressure loss and holdup. *AIChE Journal*.

[7] Laflin GC, Oglesby KD (1976). *An experimental study on the effects of flow rate, water fraction and gas-liquid ratio on air-oil-water flow in horizontal pipes [Bachelor's thesis]*.

[8] Mandhane JM, Gregory GA, Aziz K (1974). A flow pattern map for gas-liquid flow in horizontal pipes. *International Journal of Multiphase Flow*.

[9] Stapelberg H, 1 *The slug flow of oil, water and air in horizontal tubes [Ph.D. dissertation]*.

[10] Açikgöz M, França F, Lahey RT (1992). An experimental study of three-phase flow regimes. *International Journal of Multiphase Flow*.

[11] Hall ARW (1992). *Multiphase flow of oil, water and gas in horizontal pipes [Ph.D. thesis]*.

[12] Lahey RT, Acikgoz M, Franca F (1992). Global volumetric phase fractions in horizontal three-phase flows. *AIChE Journal*.

[13] Donnelly GF, Spedding PL, McBride WJ Prediction of two and three phase flow in the horizontal configuration.

[14] Ajay M (1995). *Study of two and three-phase flows in large diameter horizontal pipelines [M.S. thesis]*.

[15] Taitel Y, Barnea D, Brill JP (1995). Stratified three phase flow in pipes. *International Journal of Multiphase Flow*.

[16] Chen X, Guo L (1999). Flow patterns and pressure drop in oil-air-water three-phase flow through helically coiled tubes. *International Journal of Multiphase Flow*.

[17] Badie S, Lawrence CJ, Hewitt GF (2001). Axial viewing studies of horizontal gas-liquid flows with low liquid loading. *International Journal of Multiphase Flow*.

[18] Oddie G, Shi H, Durlofsky LJ, Aziz K, Pfeffer B, Holmes JA (2003). Experimental study of two and three phase flows in large diameter inclined pipes. *International Journal of Multiphase Flow*.

[19] Petalas N, Aziz K (2000). Mechanistic model for multiphase flow in pipes. *Journal of Canadian Petroleum Technology*.

[20] Spedding PL, Donnelly GF, Cole JS (2005). Three phase oil-water-gas horizontal co-current flow: I. Experimental and regime map. *Chemical Engineering Research and Design*.

[21] Zhang H-Q, Sarica C Unified modeling of gas/oil/water pipe flow—basic approaches and preliminary validation.

[22] Zhang H-Q, Wang Q, Sarica C, Brill JP (2003). Unified model for gas-liquid pipe flow via slug dynamics—part 1: model development. *Journal of Energy Resources Technology—Transactions of the ASME*.

[23] Wegmann A, Melke J, Rudolf von Rohr P (2007). Three phase liquid-liquid-gas flows in 5.6 mm and 7 mm inner diameter pipes. *International Journal of Multiphase Flow*.

[24] Wang S, Zhang HQ, Sarica C, Pereyra E Experimental study of high-viscosity oil/water/gas three-phase flow in horizontal and upward vertical pipes.

[25] Constant-Machado H, Leclerc J-P, Avilan E, Landaeta G, Añorga N, Capote O (2005). Flow modeling of a battery of industrial crude oil/gas separators using 113 mIn tracer experiments. *Chemical Engineering and Processing*.

[26] Jepson WP NSFI/UCRC corrosion in multiphase systems.

[27] Collier JG, Thome JR (1996). *Convective Boiling and Condensation*.

